# National patterns of paroxetine use among US Medicare patients from 2015–2020

**DOI:** 10.3389/fpsyt.2024.1399493

**Published:** 2024-07-10

**Authors:** Luke R. Cavanah, Jessica L. Goldhirsh, Leighton Y. Huey, Brian J. Piper

**Affiliations:** ^1^ Geisinger Commonwealth School of Medicine, Scranton, PA, United States; ^2^ Behavioral Health Initiative, Scranton, PA, United States; ^3^ Center for Pharmacy Innovation and Outcomes, Danville, PA, United States

**Keywords:** older adults, anticholinergic, side effects, tolerability, Beers list, potentially inappropriate medication (PIM), antidepressant, geriatric

## Abstract

**Introduction:**

Paroxetine is an older “selective” serotonin reuptake inhibitor (SSRI) that is notable for its lack of selectivity, resulting in an anticholinergic adverse-effect profile, especially among older adults (65+).

**Methods:**

Paroxetine prescription rates and costs per state were ascertained from the Medicare Specialty Utilization and Payment Data. States’ annual prescription rate, corrected per thousand Part D enrollees, outside a 95% confidence interval were considered significantly different from the average.

**Results:**

Nationally, there was a steady decrease in population-corrected paroxetine prescriptions (-34.52%) and spending (-29.55%) from 2015–2020 but a consistent, five-fold state-level difference. From 2015–2020, Kentucky (194.9, 195.3, 182.7, 165.1, 143.3, 132.5) showed significantly higher prescriptions rates relative to the national average, and Hawaii (42.1, 37.9, 34.3, 31.7, 27.7, 26.6) showed significantly lower prescription rates. North Dakota was often a frequently elevated prescriber of paroxetine (2016: 170.7, 2018: 143.3), relative to the average. Neuropsychiatry and geriatric medicine frequently prescribed the most paroxetine, relative to the number of providers in that specialty, from 2015–2020.

**Discussion:**

Despite the American Geriatrics Society’s prohibition against paroxetine use in older adults and many effective treatment alternatives, paroxetine was still commonly used in the US in this population, especially in Kentucky and North Dakota and by neuropsychiatry and geriatric medicine. These findings provide information on the specialty types and states where education and policy reform would likely have the greatest impact on improving adherence to the paroxetine prescription recommendations.

## Introduction

**Figure 1:**
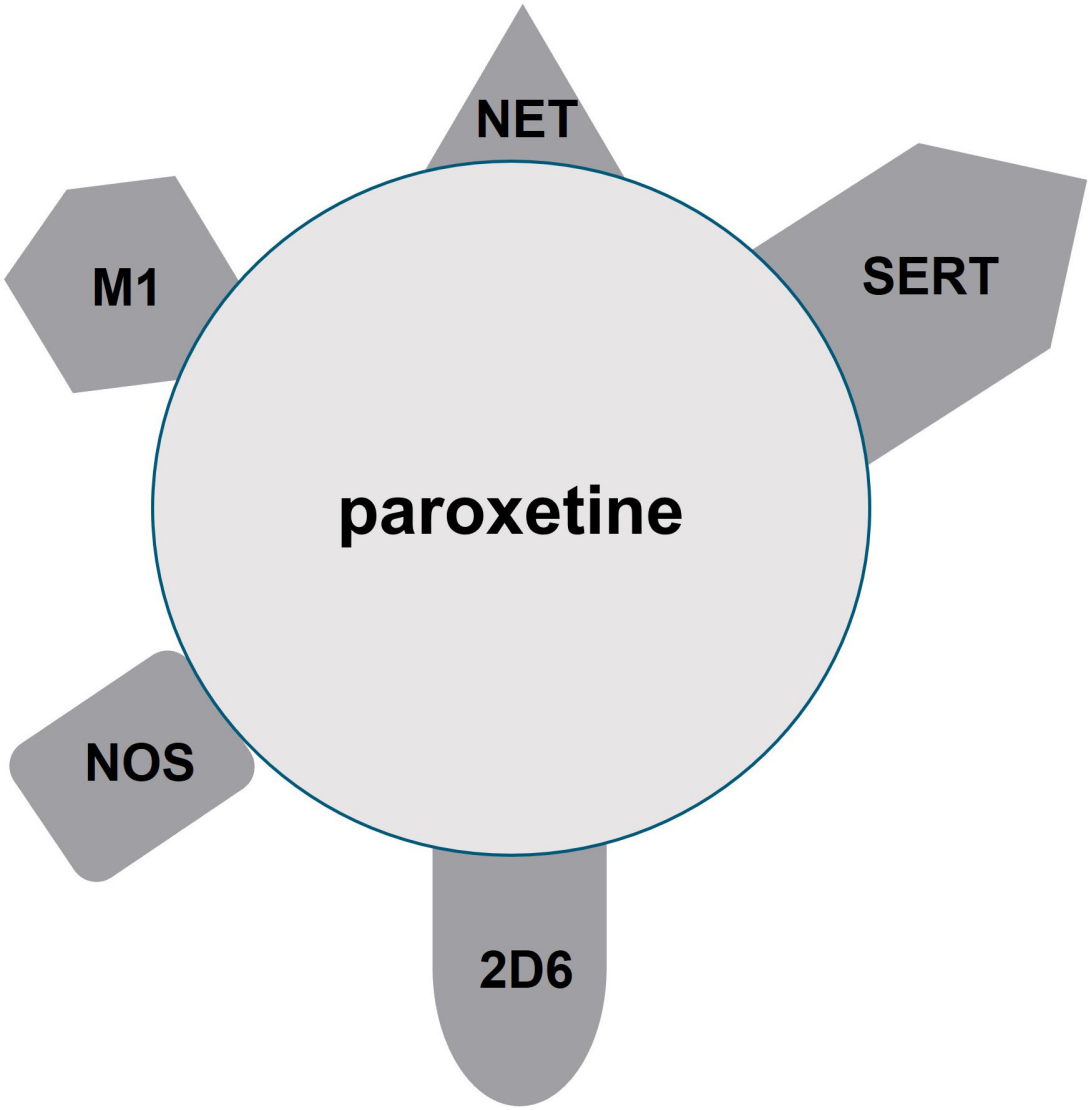
Mechanisms of action of paroxetine. Paroxetine has appreciable affinity for serotonin reuptake transporter (SERT), norepinephrine transporter (NET), central muscarinic (M_1_) receptors, nitric oxide synthase (NOS), and CYP 2D6.

“Selective” serotonin reuptake inhibitors (SSRIs), such as citalopram, escitalopram, sertraline, fluoxetine, and paroxetine, are considered first-line treatments for many psychiatric disorders, including major depressive disorder, persistent depressive disorder, generalized anxiety disorder, panic disorder, obsessive-compulsive disorder, and posttraumatic stress disorder (1–4). Despite their name, SSRIs often have activity on other chemicals, which can contribute to adverse effects (5, 6), and the unique differences in receptor profiles may help guide a clinician’s preference for a particular clinical situation or patient population. One SSRI that is particularly infamous for its nonselective action on other neurotransmitters and liver enzymes is paroxetine (7). Paroxetine was first marketed in the US in 1992, and it is indicated for use in depression, obsessive-compulsive disorder, panic disorder, social anxiety disorder, and generalized anxiety disorder. Paroxetine is available in generic and brand names (Paxil ^®^, Pexeva ^®^, Brisdelle^®^). **Figure 1** illustrates that in addition to its notable target at the serotonin reuptake transporter (SERT), it has an appreciable affinity for the norepinephrine transporter (NET), central muscarinic (M_1_) receptors, nitric oxide synthase (NOS) (8), and CYP 2D6 (6, 9). Some research also suggests paroxetine additionally has activity at CYP 3A4 (10). As a consequence of the lack of specificity, paroxetine, compared to other SSRIs, results in increased sedation, constipation, sexual dysfunction, discontinuation syndrome, and weight gain (11).

Compared to all SSRI medications, paroxetine has the highest affinity for the M_1_ receptor (*K_i_* = 76 nM). Consequently, paroxetine use is associated with classical anticholinergic side effects, such as constipation, urinary retention, increased intraocular pressure, blurred vision, dry mouth, dry eyes, flushing, and hyperthermia (4, 9, 12, 13). Older adult populations are particularly at risk of anticholinergic effects, so the use of anticholinergic medications, such as paroxetine, in older adults should be reduced when clinically appropriate, especially in those suffering from dementia (14–16). Furthermore, anticholinergic drugs have been demonstrated to contribute to cognitive decline and dementia in older adults (17). Syndrome of inappropriate anti-diuretic hormone (SIADH) is also a well-known, concerning adverse effect of various SSRIs, and older age is a risk factor of SSRI-induced SIADH due to age-related changes in renal functioning (12, 18).

In addition to paroxetine’s lack of receptor selectivity with an appreciable affinity for the norepinephrine transporter and nitric oxide (8), it also has many medication interactions due to action on the CYP450 superfamily (7). Paroxetine is a strong inhibitor of the P450 3A4 isoenzyme, which metabolizes approximately 50% of prescribed drugs (10). Moreover, paroxetine is the strongest inhibitor of P450 2D6 isoenzyme (*K_i_* = 0.065–4.65 μM) of all antidepressants (6, 9). CYP2D metabolizes many medications, such as antipsychotics, tricyclic antidepressants, class IC antiarrhythmics, β-adrenergic agents, trazodone, and dextromethorphan (19). Older adults tend to take more medications, with an average of six to eight (20–22), increasing the risk of drug-drug interactions. What is more concerning is that there is evidence of growing rates of polypharmacy (23), further emphasizing the importance of re-evaluating prescribing practices in older adults and paying particular attention to those medications that have many possible drug-drug interactions.

Adverse drug events in older adults are certainly not unique to paroxetine: approximately 15% of hospitalizations of this population are secondary to an adverse drug event (24); this value is double for those over 75 (25, 26). In the outpatient setting, it is estimated that approximately 30% of older adults are taking potentially inappropriate medications (PIMs) (27). The American Geriatric Society (AGS) Beers Criteria is an explicit list of medications, labeled as PIMs, that should generally be avoided by older adults in most circumstances, or in certain disorders/conditions specified by the AGS (28–30). Approximately 30–50% of adverse drug events are preventable (25, 31). Clearly, minimizing such adverse events has significant health and economic consequences on both a micro and a macro scale.

While the guidelines from the American Psychiatric Association recommend SSRIs, including paroxetine, as first-line treatments for various depressive and anxiety disorders (2, 3, 32), there is growing consensus that paroxetine is less preferable than other SSRIs for older adults with these conditions (28–30). Many leading professional organizations in the field of geriatrics, such as the AGS, who are clear that paroxetine is a potentially higher risk medication that should be avoided in older adults when possible, labeling paroxetine as a PIM. According to the AGS Beers Criteria, there is high quality evidence indicating that paroxetine is strongly anticholinergic and has an unfavorable likelihood of causing sedation and orthostatic hypotension and thus, it is strongly recommended that paroxetine should be avoided in older adults whenever possible (28–30). There are several safer alternative therapeutic options for older adults, such as citalopram, escitalopram, sertraline, venlafaxine, mirtazapine, and bupropion (33).

A recent investigation demonstrated concerningly high use of the nonbenzodiazepine hypnotics, a class of psychiatric medications considered as PIMs by the AGS among Medicare patients (34). Further, it was noted that such prescription patterns displayed significant variation among states and specialties (34). However, no study to date has examined such prescribing patterns of paroxetine in this population. The purpose of this study was to examine patterns in paroxetine prescription rates throughout the United States among Medicare patients. Specifically, this study was aimed at characterizing the chronological, geographical, and specialty patterns of paroxetine prescriptions from 2015–2020. This study has important implications for education and policy reform regarding paroxetine use in older adults.

## Methods

### Data source

Utilization and cost data was extracted from Medicare Specialty Utilization and Payment Data (35). These publicly available datasets include information on medications prescribed under the Medicare Part D Prescription Drug Program. We analyzed the dataset ‘Medicare Part D Prescribers – by Geography and Drug’ to assess national and state annual prescription rates and costs. We analyzed the dataset ‘Medicare Part D Prescribers – by Provider and Drug’ to assess differences in prescription patterns among specialties. Both datasets include the variables of the geographic location of the prescribers, the trademarked name of the medication filled (i.e., brand name), chemical ingredient of the drug (i.e., generic name), total claims, total 30-day fills, aggregate drug cost paid for associated claims, and total number of distinct Part D beneficiaries with at least one claim. The ‘Medicare Part D Prescribers – by Geography and Drug’ dataset aggregates data by drug and state/territory, suppressing when total claims are less than 11. The ‘Medicare Part D Prescribers – by Provider and Drug’ dataset aggregates data by individual prescribers, noting their names, city, state, and type (e.g., specialty), suppressing when total claims are less than 11. The number of providers per specialty was obtained from Medicare Physician and Other Practitioners by Specialty and Service (36). Medicare Specialty Utilization and Payment Data has been used in other pharmacoepidemiology reports (34, 37, 38).

### Population and exposure

The Medicare program covered 18.4% of the US population in 2020 (39), and 76.0% have Part D coverage (40). Further, it covers 94% of non-institutionalized persons age 65 and older (41). This includes 61.5 million people of which 86.8% were greater than or equal to age 65, and 13.2% were disabled. Medicare claims data from 2015–2020 with generic name including “paroxetine” (e.g., paroxetine hcl, paroxetine mesylate) were included in the analysis. The database derives the generic names from National Drug Codes provided in Prescription Drug Event data. Data for which the prescriber geographic level was “national” was excluded, so that data from the fifty states and District of Columbia were not double counted. Further, data for which the prescriber geographic description was designated as US territories, Armed Force areas, Unknown and Foreign Country were excluded. Prescriptions reflected prevalent prescriptions: it included original prescriptions and refills. Costs data reflects aggregate drug cost paid for all associated claims. Specifically, the value for cost includes ingredient cost, dispensing fee, sales tax, and is based on the amounts paid by the Part D plan, Medicare beneficiary, government subsidies, and any other third-party payers.

### Procedures

National and state-level annual (2015–2020) paroxetine prescription rates and costs were obtained for Medicare Part D patients. We evaluated the Medicare Specialty Utilization and Payment Data for paroxetine prescription rates for all fifty states and the District of Columbia (35). This dataset provides information on prescription medications prescribed by healthcare professionals that are paid for by the Medicare Part D Prescription Drug Program. Prescription rates were reported per thousand Medicare Part D enrollees to account for differences in the population of different states, and spending rates were reported in dollars per enrollee. Procedures were approved as exempt by the Geisinger IRB.

### Data analysis

National and state-level patterns in the number of prescriptions of generic, brand, and their sum were compared for paroxetine. One-sample z-tests were conducted to determine whether annual prescription rates for each individual state were significantly different from the average across all the states for a respective year. States with population-corrected prescriptions outside 1.5 and 1.96 standard deviations from the state average of that year were also identified. The ratio of the number of prescriptions, corrected for the number of enrollees, for the highest and lowest states was calculated as an index of state-level disparities. The percent of total Medicare spending for generic versus brand was also calculated.

To understand specialty-type variations, ratios were calculated. Percent of paroxetine prescriptions from a particular specialty relative to all specialties was calculated. The aforesaid percent was divided by the percent of providers in Medicare who belong to a particular specialty. Ratios greater than 1.0 suggested the specialty was overrepresented, and ratios under 1.0 suggested the specialty was underrepresented for paroxetine prescriptions. Specialties for whom taxonomy codes could not be mapped to a Medicare specialty code were excluded. The methods employed in this investigation have been used to assess chronologic, geographic, and specialty prescribing patterns of other medications among Medicare patients (34, 37, 38). Data was analyzed using Excel and figures were constructed using GraphPad Prism and Heatmapper (42).

## Results

**Figure 2:**
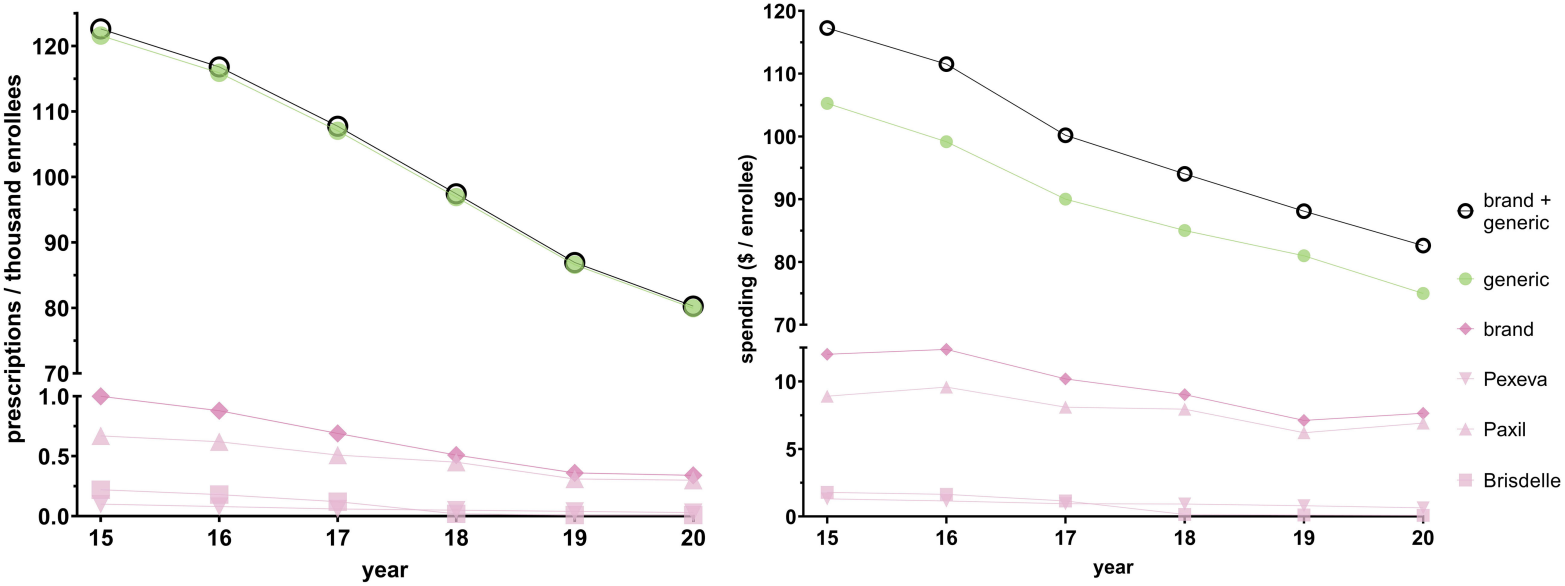
National Medicare prescription rates (left) and Medicare spending (right) for paroxetine for 2015–2020.

**Figure 2**; **Supplementary Table 1** shows that population-corrected national paroxetine use and spending gradually decreased from 2015 to 2020. There were 122.61 prescriptions per thousand Medicare enrollees in 2015. This decreased by 34.52% to 80.28 prescriptions per thousand Medicare enrollees in 2020. There was $117.27 spent per enrollee in 2015. This value decreased by 29.55% to $82.62 per enrollee in 2020. Generic paroxetine consistently constituted ≥ 99.2% of all prescriptions, and increased by 0.5%, when spending on generics consistently constituted around 90% of all paroxetine spending.

**Figure 3:**
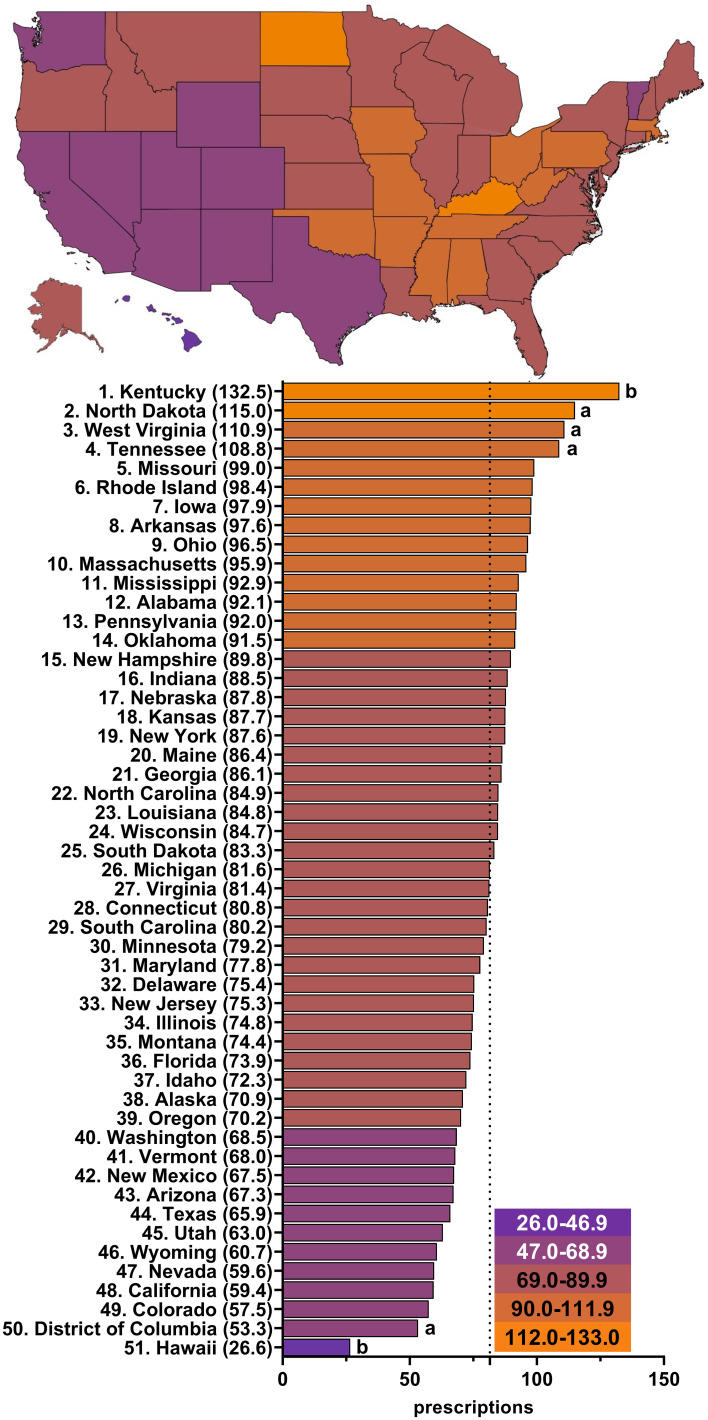
Paroxetine prescriptions per thousand Medicare Part D enrollees heatmap (top) and population-corrected prescription rate per state (bottom) in 2020. ^a^ indicates >1.50 SD (17.4) from the mean (81.5), denoted by the dotted line. ^b^ indicates >1.96 SD from the mean.

**Figure 3**; **Supplementary Figures 1**-**5**; **Supplementary Table 2** show wide state-level variation in 2015 (4.6 fold), 2016 (5.2 fold), 2017 (5.3 fold), 2018 (5.2 fold), 2019 (5.2 fold), and 2020 (5.0 fold). Kentucky was always the highest prescribing state with a significantly greater number of prescriptions than the mean number of state prescriptions in all years examined. North Dakota was the second highest prescribing state, except for 2015 and 2017, and had a significantly higher number of paroxetine prescriptions than the mean number of state prescriptions in 2016, 2018, and 2019. Alaska had significantly more paroxetine prescriptions than average in 2015. On the other end of the spectrum, Hawaii, the lowest prescribing state, had a significantly lower number of prescriptions than the mean number of state prescriptions in all years examined. The District of Columbia, consistently the second or third lowest prescribing municipality, prescribed significantly less paroxetine than other states in 2015, 2016, and 2018.

**Figure 4:**
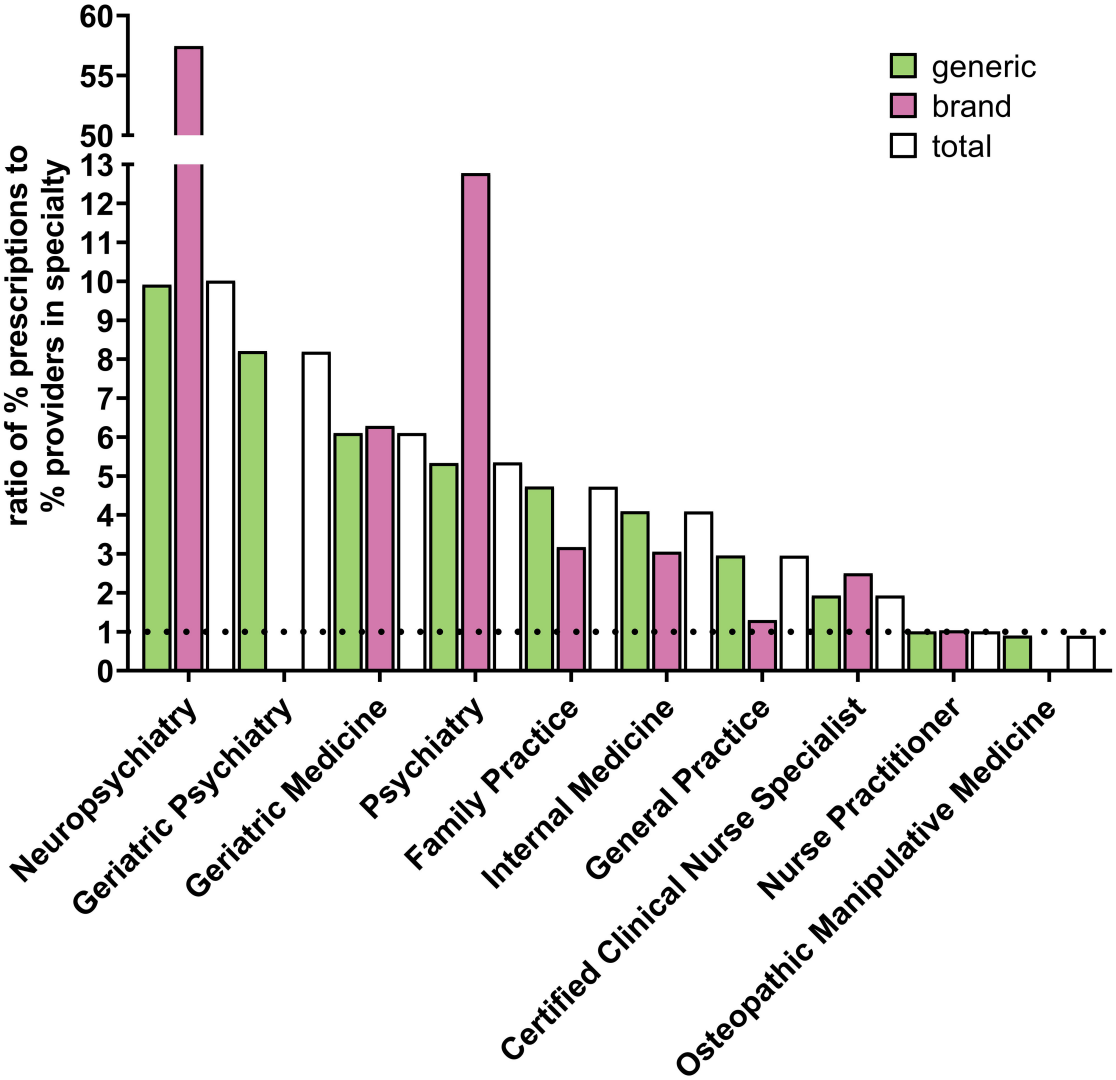
*Specialty types that prescribe the most paroxetine to Medicare Part D enrollees for 2020.* Specialty types that had the highest ratio of percent of paroxetine prescriptions to percent of providers in Medicare who belong to that respective specialty. Dotted line denotes ratio of 1.0.

**Figure 4**; **Supplementary Figure 2**; **Supplementary Table 3** shows the specialties with the highest ratios of percent of paroxetine prescriptions to percent of practitioners in Medicare who belong to that respective specialty. For all years examined, neuropsychiatry, geriatric medicine, geriatric psychiatry, psychiatry, family practice, internal medicine, general practice, and certified clinical nurse specialists had ratios above 1, with neuropsychiatry and geriatric medicine consistently having the highest ratios, except for geriatric psychiatry having a higher ratio than geriatric medicine in 2020. Neuropsychiatry and psychiatry notably and consistently had the highest ratios for brand paroxetine prescriptions for all years examined.

## Discussion

We found appreciable use of paroxetine nationally (123 prescriptions/1,000 enrollees) among US Medicare patients in 2015. It is encouraging that the data in this study show that paroxetine use in a population who is not generally recommended has been in a steady decline for the generic formulations. Similarly, branded formulations, although uncommon (43), also declined. Continued recommendations against the use of paroxetine among older adults (29), as well as increased therapeutic alternatives likely has contributed to the decline so far observed and serves as potential means for the decline to continue (7, 11, 33). Previously mixed recommendations (28) on the use of paroxetine in older adults may be contributing to its abatement among older adults in recent years. It is worth noting that there are individual considerations that may make the evidence against paroxetine not applicable to a given provider-patient relationship.

Kentucky was consistently an elevated prescriber of paroxetine, and North Dakota was often an elevated prescriber of paroxetine, suggesting these are the states that appear to have the largest potential benefit to the introduction and/or improvement of education and/or policy related to the recommended uses of paroxetine. Similarly, the specialties that were found to be overrepresented in paroxetine prescriptions and therefore have the most potential benefit from education and policy changes related to paroxetine use among older adults are neuropsychiatry, geriatric medicine, geriatric psychiatry, psychiatry, family practice, internal medicine, general practice, and certified clinical nurse specialists. The high number of specialties found to be overrepresented in paroxetine prescribing likely reflects the frequency by which SSRIs are prescribed by numerous specialties. Further, it suggests that interventions aimed at optimizing paroxetine use would likely yield limited benefit by focusing on one or few specialties. More research needs to be done to understand the geographic and specialty variation seen in this study to draw conclusions as to potential causes for the unexpected heterogeneity.

Regardless, the persistent relatively frequent use of paroxetine in this population is concerning, especially given the existence of safer alternatives (28, 29, 33). Even more, although the spending on paroxetine by older adults illustrated in **Figure 2** averages around a sizable $100 per enrollee, it does not capture the inpatient expenditures and treatments that older adults may need as consequence of inappropriate use of paroxetine, which impacts the higher spending in high-risk populations more than pharmaceutical costs (44). With the continued prevalent use of paroxetine in older adults, implementation of ongoing computerized reminder systems may be worth consideration, as well as improving prescriber education and feedback on geriatric pharmacotherapy (45, 46). Other strategies that could be used to improve prescribing practices of paroxetine could be the integration of clinical pharmacists in medication reviews in older adult patients, which has been shown to reduce total number of medications, PIMs, and potential drug-drug interactions (46, 47). Moreover, the overall high frequency of adverse drug events (24–26) and use of PIMs (27) in older adults suggests that routine review of this population’s medication regimen with a standardized tool, such as Beers (28–30) or STOPP/START (48), would likely have a substantial impact on improving the healthcare outcomes of the age demographic that uses most health care resources (49).

The observed decline in paroxetine utilization and expenditure among older adults may be explained by the advent of newer, more efficacious and tolerable antidepressants, so further study of the pharmacoepidemiology of these alternatives would be helpful. Thus, the decreasing pattern in paroxetine use found in this study may merely reflect the general decrease in use of paroxetine, not due to recommendations to avoid paroxetine use in older adults. Future studies would be needed to determine if the decrease in paroxetine use observed in this study is unique to older adults or if it is consistent across other age demographics.

Although the focus of this study was understanding paroxetine use among older adults, there are many other medications, drug interactions, and prescribing practices such as polypharmacy that contribute to serious adverse events in older adults. Future directions may include characterizing the pharmacoepidemiology of these other high-risk medications in older adults, as well as monitoring polypharmacy among older adults with paroxetine and other drugs. Further, understanding the clinical consequences of polypharmacy with specific medications, such as paroxetine, is an important endeavor. Additionally, examining how the prescribing patterns of these high-risk medications has changed as result of the implementation of different clinical tools, such as the Beers (30), STOPP and START (48) criteria would be meaningful.

## Limitations

Although the Medicare program serves 94% of non-institutionalized persons age 65 and older, some limitations of this study are noteworthy (41). First, about one seventh of those who have Medicare are under the age of 65, and one-quarter of people with Medicare do not have Part D coverage (40). Second, further study with other databases will be necessary to characterize whether paroxetine was prescribed for anxiety disorders, major depression, or an off-label indication (e.g., sleep disturbance or sedation in nursing home residents), as this information is not available in the Medicare database analyzed. Third, this database does not provide the age and sex of the specific patients for which the medication was prescribed. Fourth, the current database does not provide information on the other medications a particular patient prescribed paroxetine may have been taking, limiting conclusions that can be drawn regarding potential paroxetine-mediated drug-drug interactions. Fifth, it is worth noting despite significant evidence suggesting paroxetine has significantly higher risk when used in the older adult population, some studies have demonstrated no important cognitive adverse effects in this population (50, 51).

## Conclusion

In conclusion, paroxetine use among the Medicare population has remained high, albeit steadily decreasing from 2015–2020, despite the American Geriatrics Society identifying paroxetine as a potentially higher risk medication that should be avoided in older adults when possible. Further, there was a consistent five-fold state-variation in population-corrected paroxetine prescriptions, with Kentucky consistently prescribing more than average, North Dakota often prescribing more than average, and Hawaii consistently prescribing less than average. Many specialties were found to be overrepresented in number of paroxetine prescriptions. Future studies should explore the reasons for the decline in paroxetine use, the pronounced state-level differences, and the specialty variation in paroxetine use among the Medicare population.

## Data availability statement

Publicly available datasets were analyzed in this study. This data can be found here: https://data.cms.gov/provider-summary-by-type-of-service/medicare-part-d-prescribers, https://data.cms.gov/provider-summary-by-type-of-service/medicare-physician-other-practitioners/medicare-physician-other-practitioners-by-provider-and-service.

## Ethics statement

The studies involving humans were approved by Geisinger Institutional Review Board. The studies were conducted in accordance with the local legislation and institutional requirements. Written informed consent for participation was not required from the participants or the participants’ legal guardians/next of kin in accordance with the national legislation and institutional requirements.

## Author contributions

LC: Data curation, Formal analysis, Investigation, Methodology, Resources, Visualization, Writing – original draft, Writing – review & editing. JG: Conceptualization, Writing – review & editing. LH: Writing – review & editing. BP: Conceptualization, Funding acquisition, Investigation, Methodology, Resources, Software, Supervision, Writing – review & editing.
