# Supplementary material for: National patterns of paroxetine use among US Medicare patients from 2015–2020
Source: Front Psychiatry. 2024 Jul 10;15:1399493. doi: 10.3389/fpsyt.2024.1399493 (PMC11266311; doi:10.3389/fpsyt.2024.1399493)
Supplement: Supplementary file 1 [file DataSheet_1.docx]

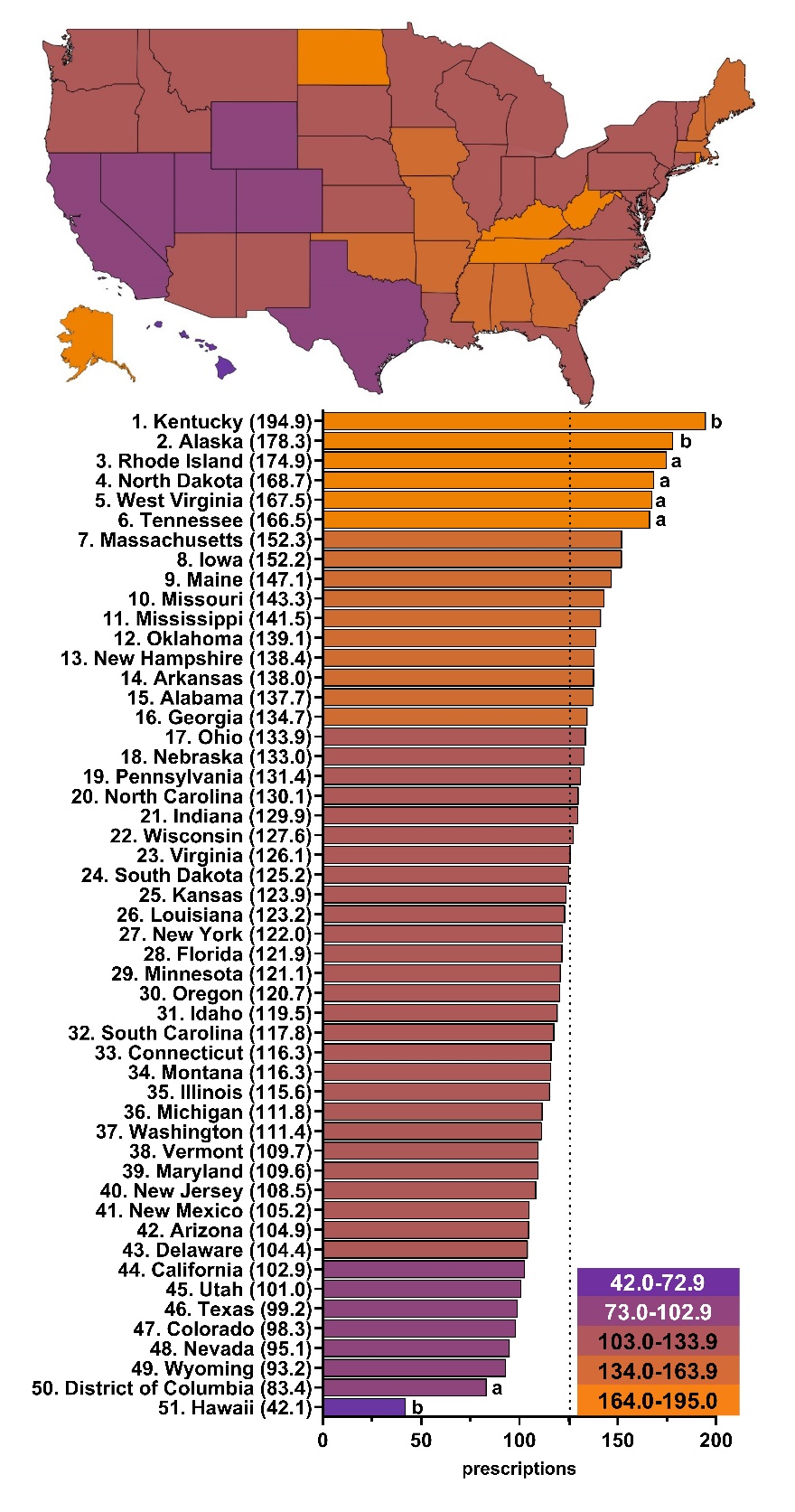


**Supplemental Figure 1.** Paroxetine prescriptions per thousand Medicare Part D enrollees heatmap (top) and population-corrected prescription rate per state (bottom) in 2015. ^a^ indicates >1.50 SD (26.2) from the mean (125.7), denoted by the dotted line. ^b^ indicates >1.96 SD from the mean.

**
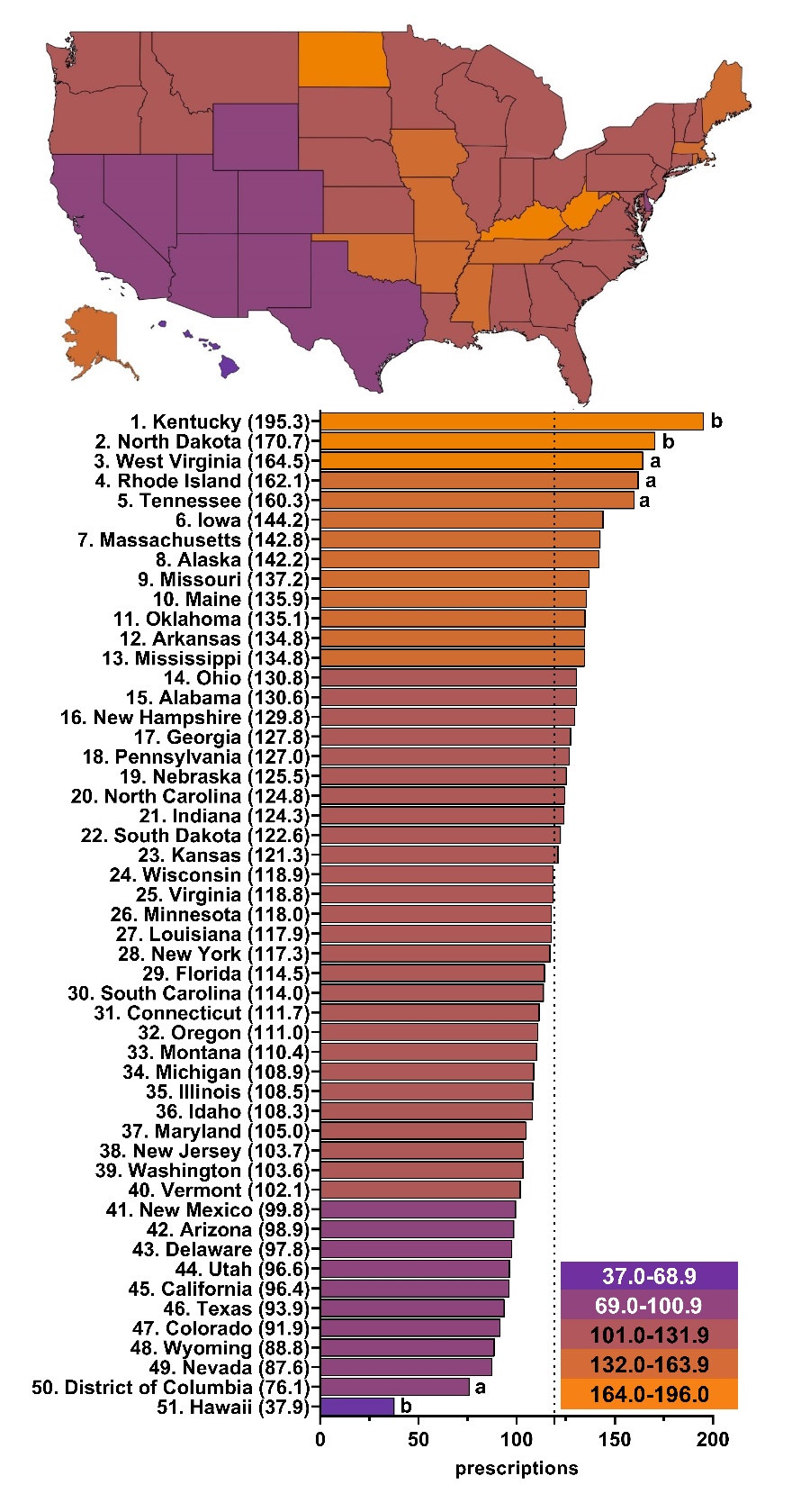
**

**Supplemental Figure 2.** Paroxetine prescriptions per thousand Medicare Part D enrollees heatmap (top) and population-corrected prescription rate per state (bottom) in 2016. ^a^ indicates >1.50 SD (25.6) from the mean (119.3), denoted by the dotted line. ^b^ indicates >1.96 SD from the mean.

**
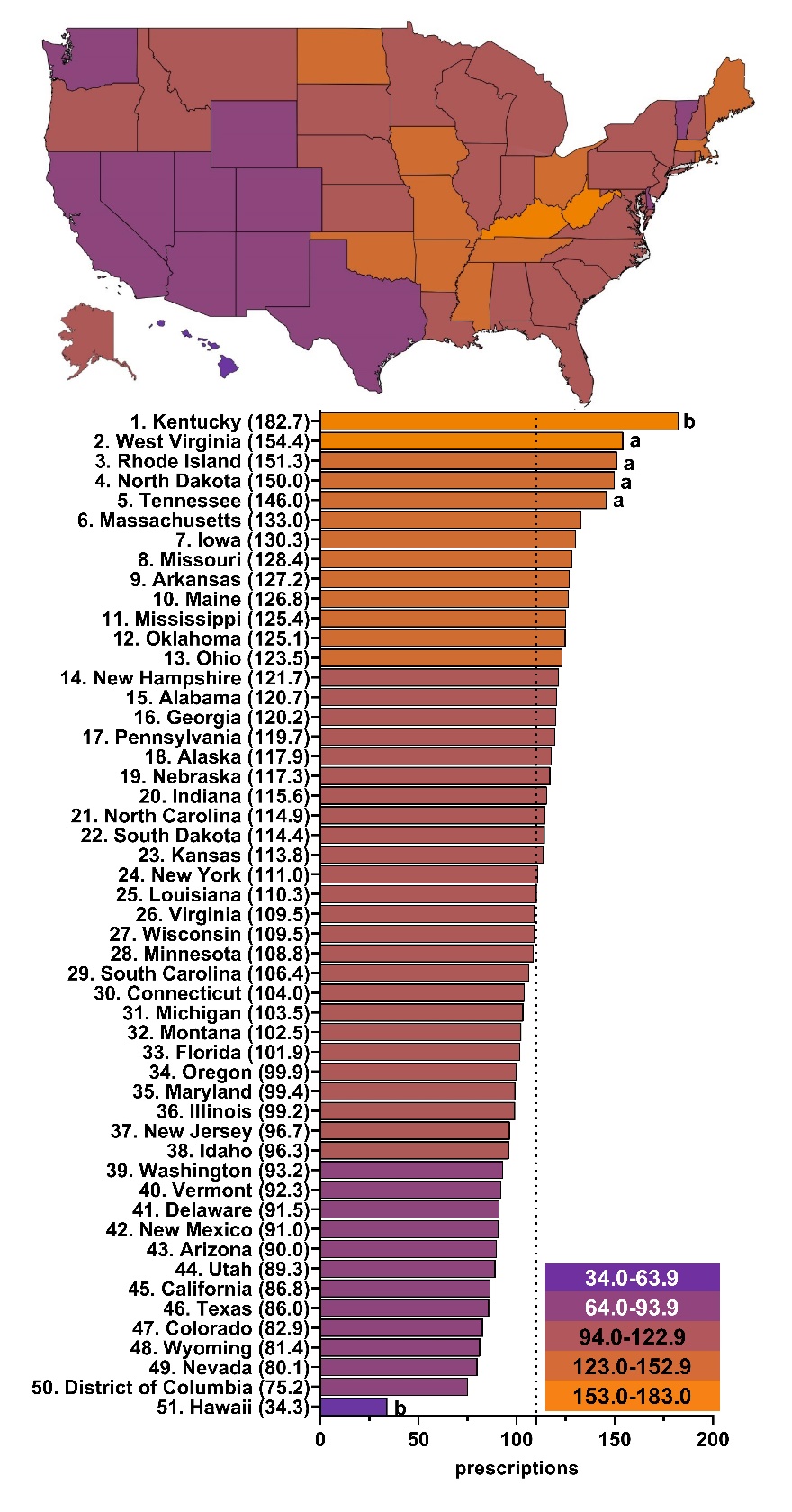
**

**Supplemental Figure 3.** Paroxetine prescriptions per thousand Medicare Part D enrollees heatmap (top) and population-corrected prescription rate per state (bottom) in 2017. ^a^ indicates >1.50 SD (23.7) from the mean (110.1), denoted by the dotted line. ^b^ indicates >1.96 SD from the mean.

**
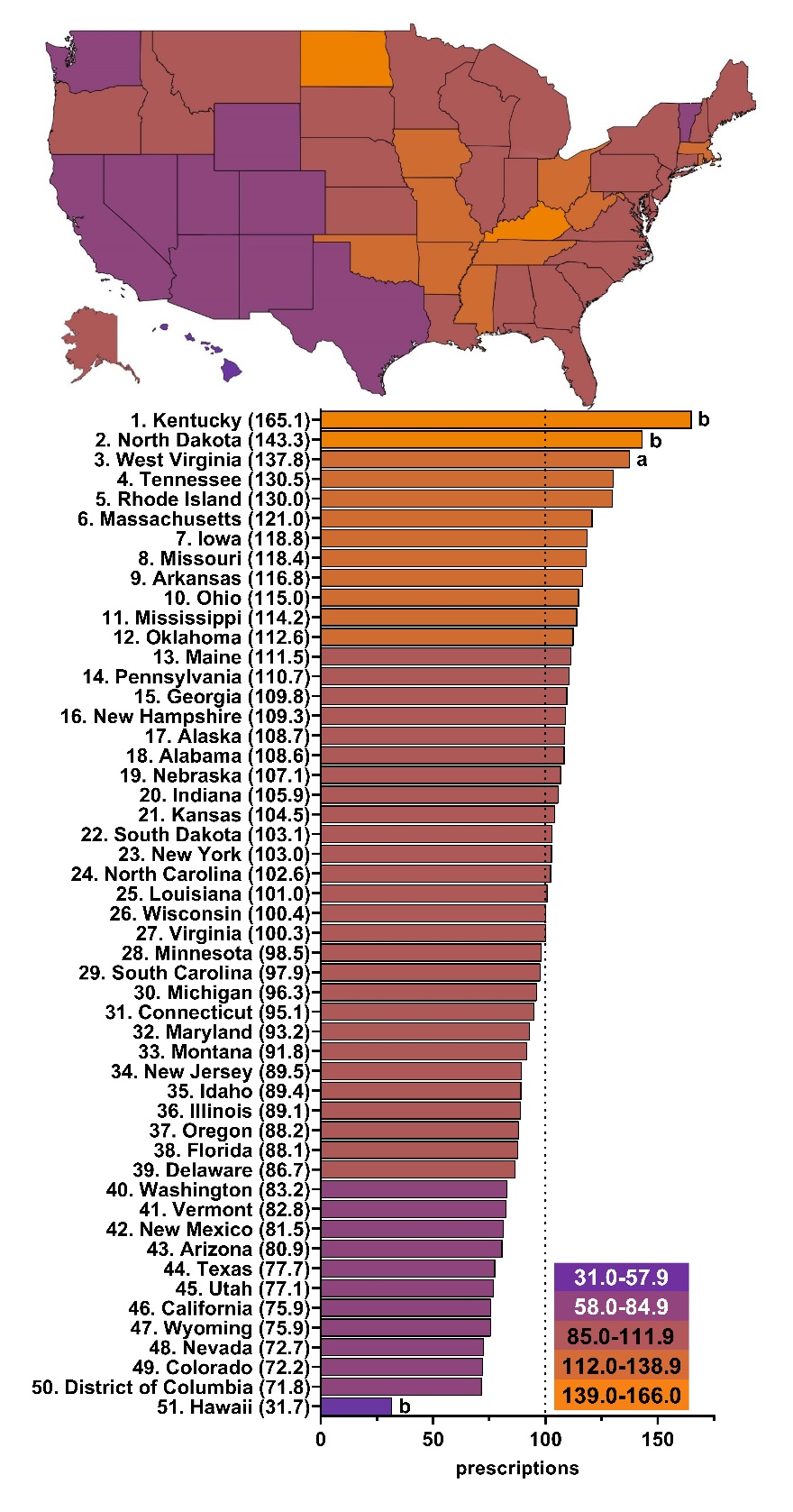
**

**Supplemental Figure 4.** Paroxetine prescriptions per thousand Medicare Part D enrollees heatmap (top) and population-corrected prescription rate per state (bottom) in 2018. ^a^ indicates >1.50 SD (21.5) from the mean (99.9), denoted by the dotted line. ^b^ indicates >1.96 SD from the mean.

**
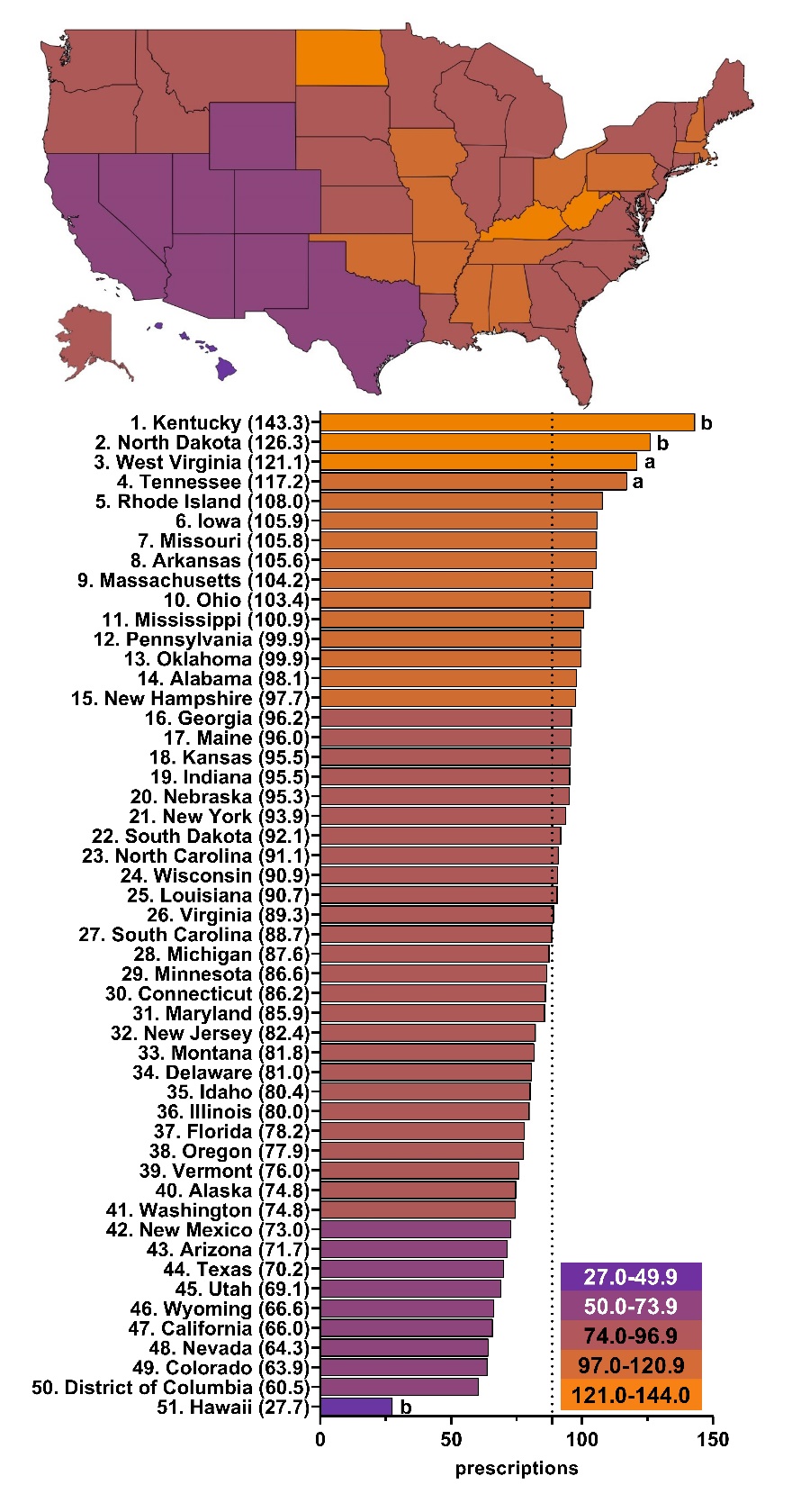
**

**Supplemental Figure 5.** Paroxetine prescriptions per thousand Medicare Part D enrollees heatmap (top) and population-corrected prescription rate per state (bottom) in 2019. ^a^ indicates >1.50 SD (18.7) from the mean (88.6), denoted by the dotted line. ^b^ indicates >1.96 SD from the mean.

**
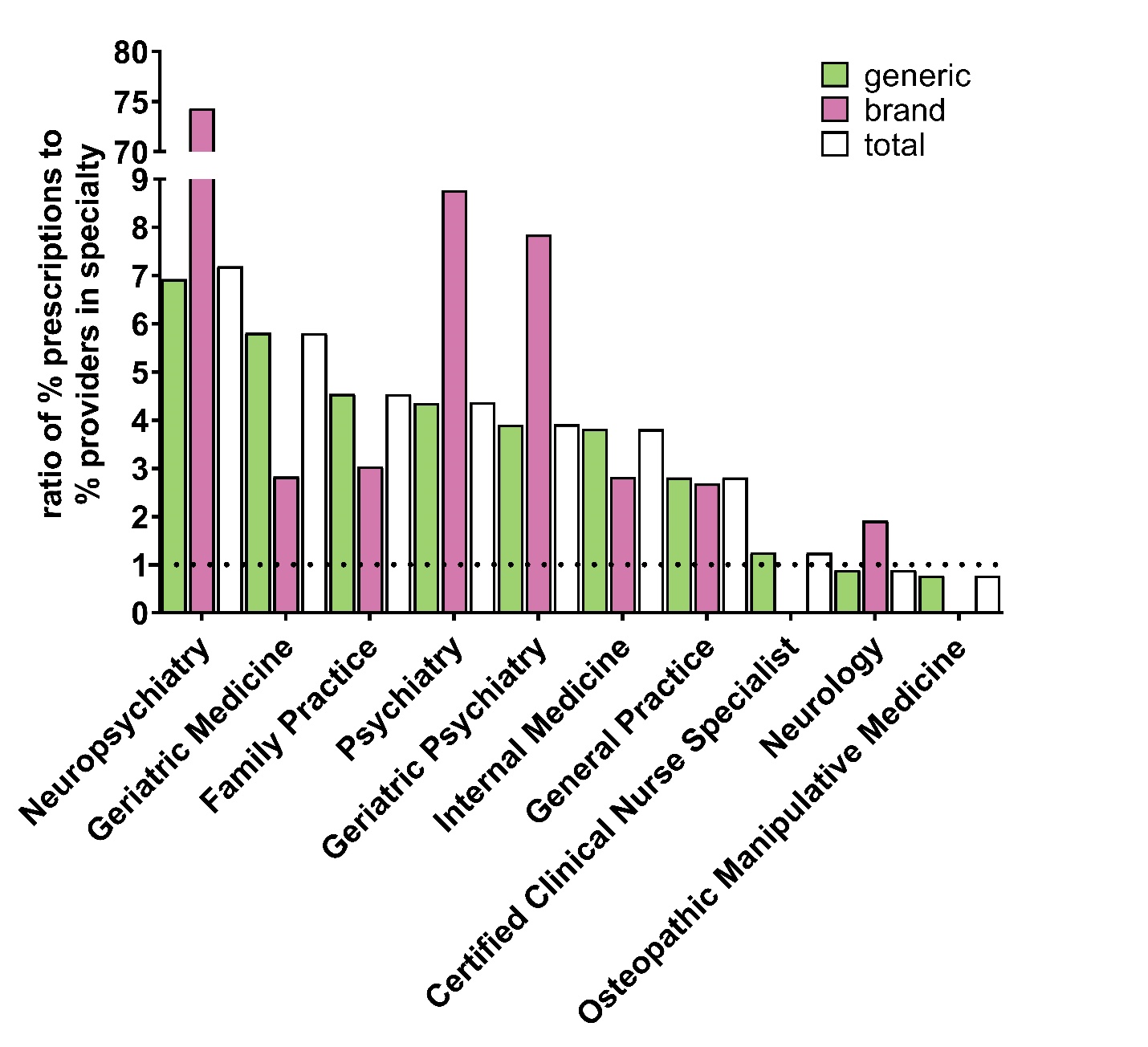
**

**Supplemental Figure 6.** *Specialty types that prescribe the most paroxetine to Medicare Part D enrollees for 2015.* Specialty types that had the highest ratio of percent of paroxetine prescriptions to percent of providers in Medicare who belong to that respective specialty. Dotted line denotes ratio of 1.0.


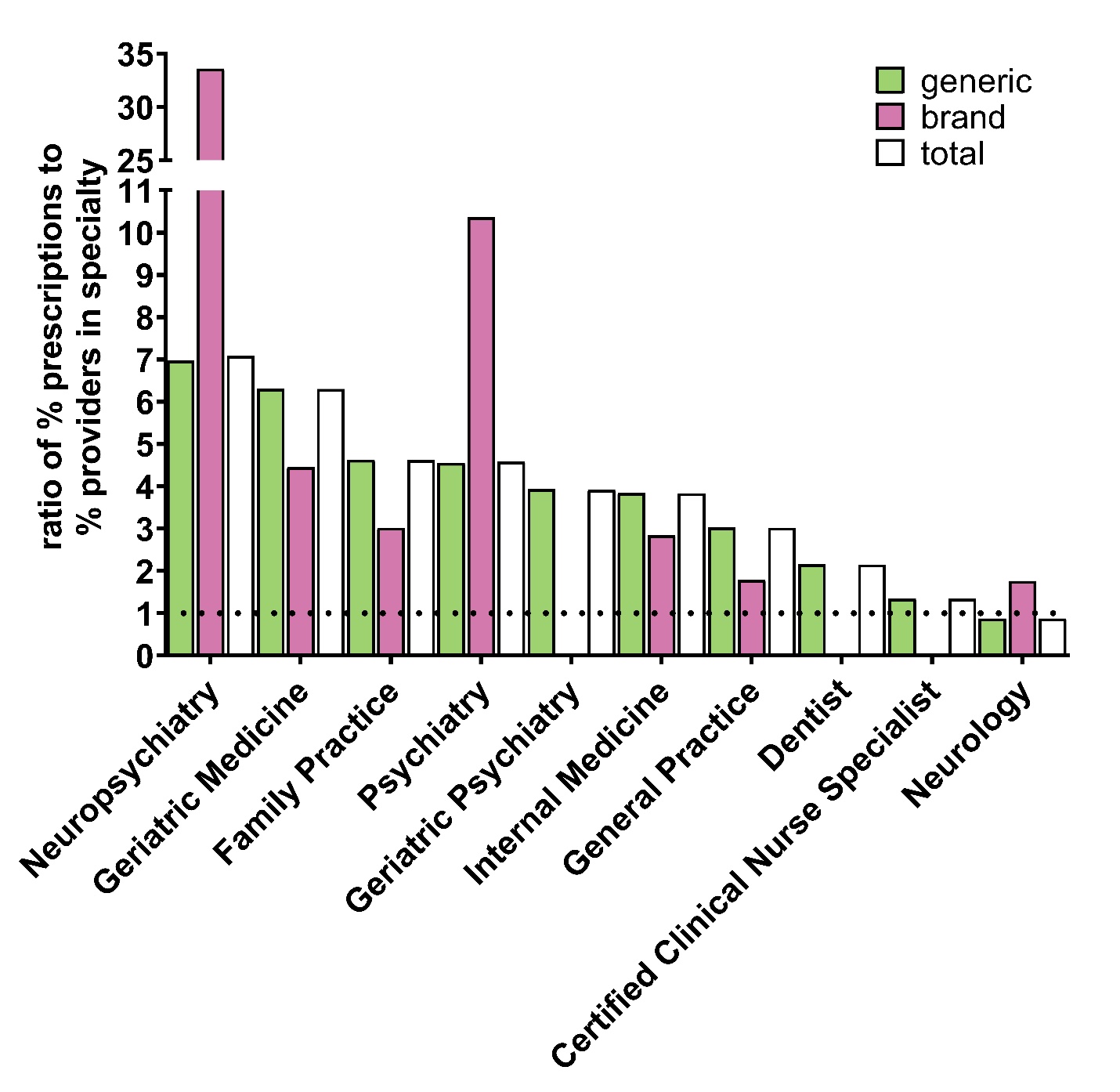


**Supplemental Figure 7.** *Specialty types that prescribe the most paroxetine to Medicare Part D enrollees for 2016.* Specialty types that had the highest ratio of percent of paroxetine prescriptions to percent of providers in Medicare who belong to that respective specialty. Dotted line denotes ratio of 1.0.


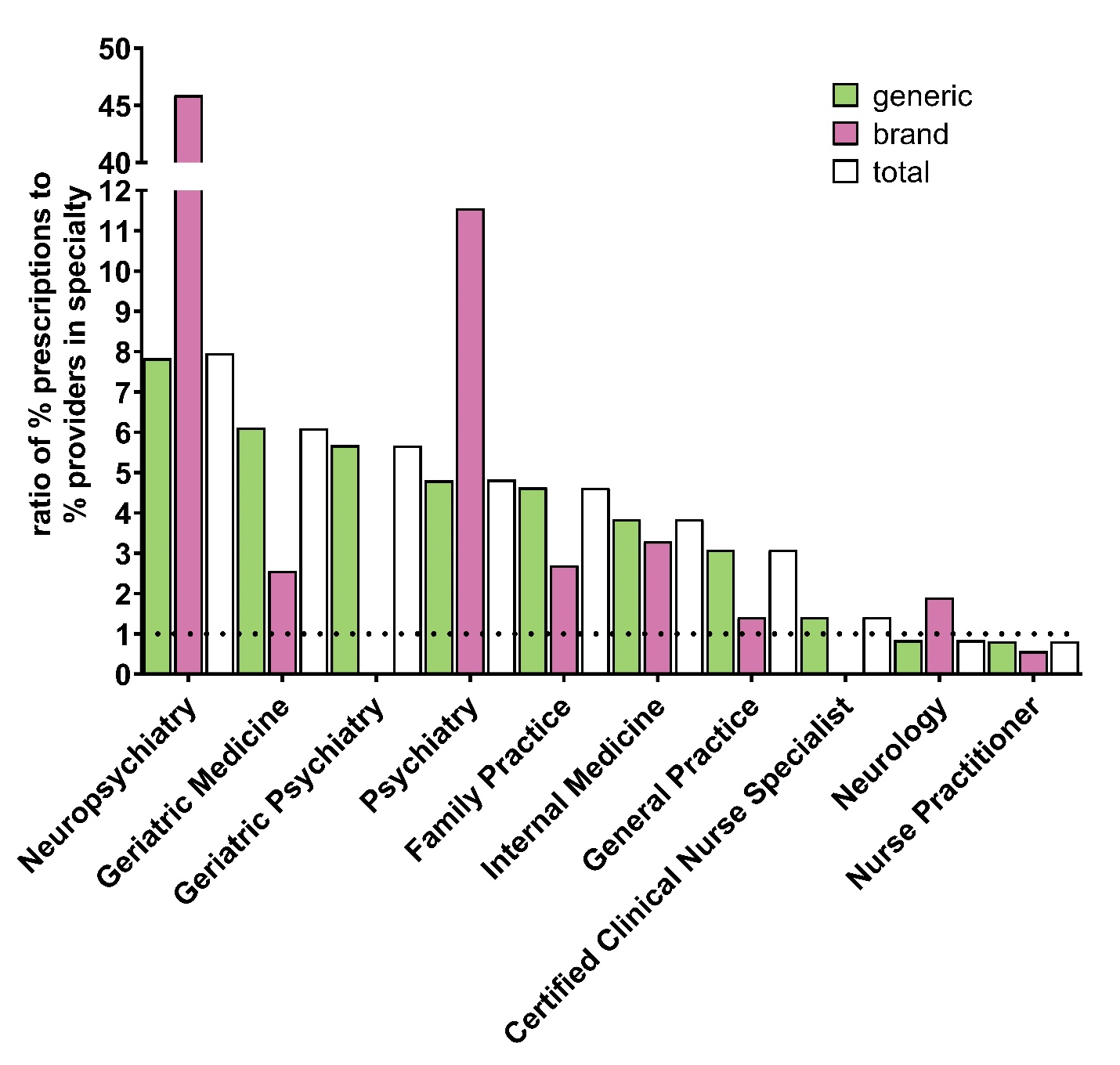


**Supplemental Figure 8.** *Specialty types that prescribe the most paroxetine to Medicare Part D enrollees for 2017.* Specialty types that had the highest ratio of percent of paroxetine prescriptions to percent of providers in Medicare who belong to that respective specialty. Dotted line denotes ratio of 1.0.

**
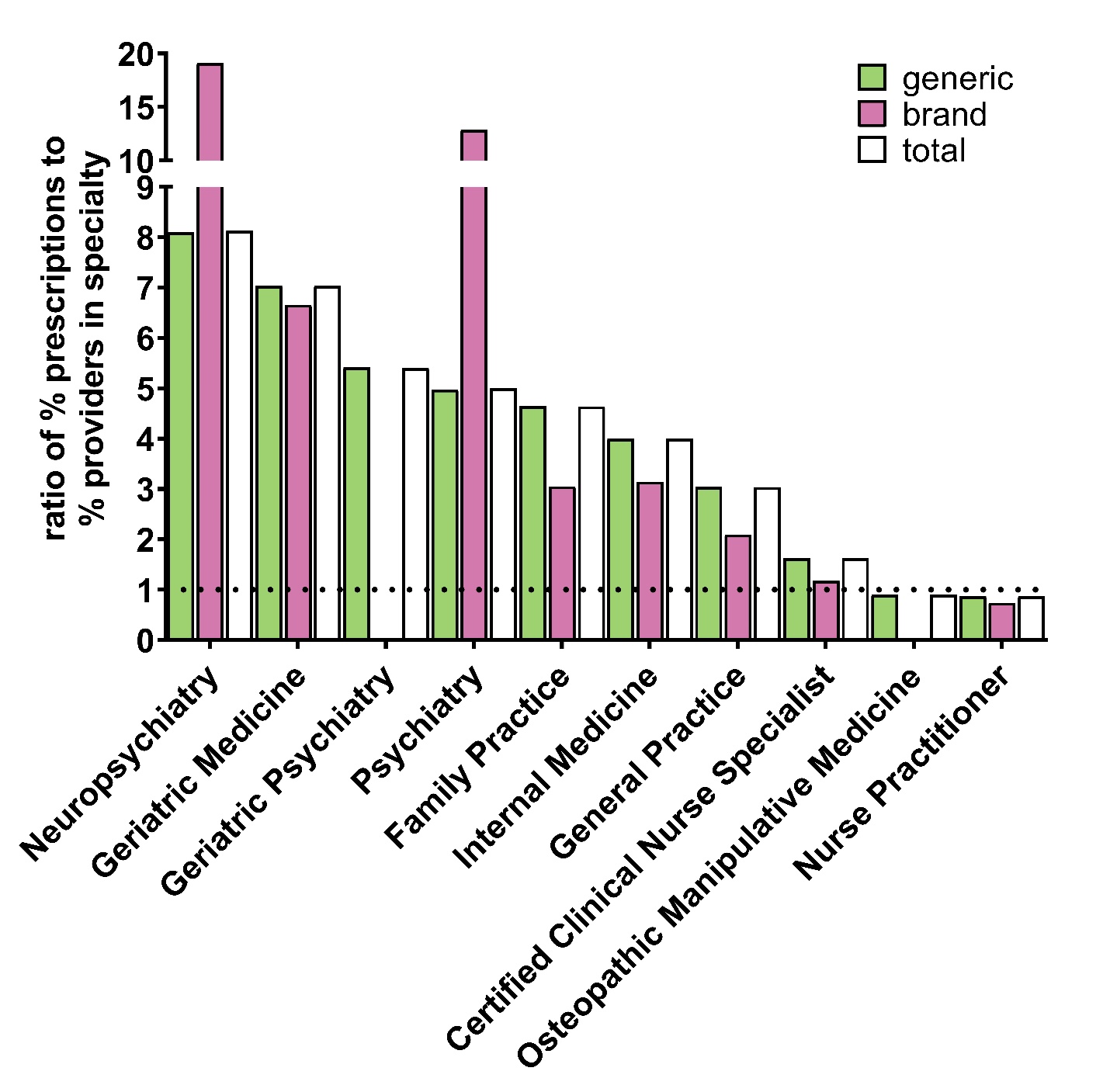
**

**Supplemental Figure 9.** *Specialty types that prescribe the most paroxetine to Medicare Part D enrollees for 2018.* Specialty types that had the highest ratio of percent of paroxetine prescriptions to percent of providers in Medicare who belong to that respective specialty. Dotted line denotes ratio of 1.0.

**
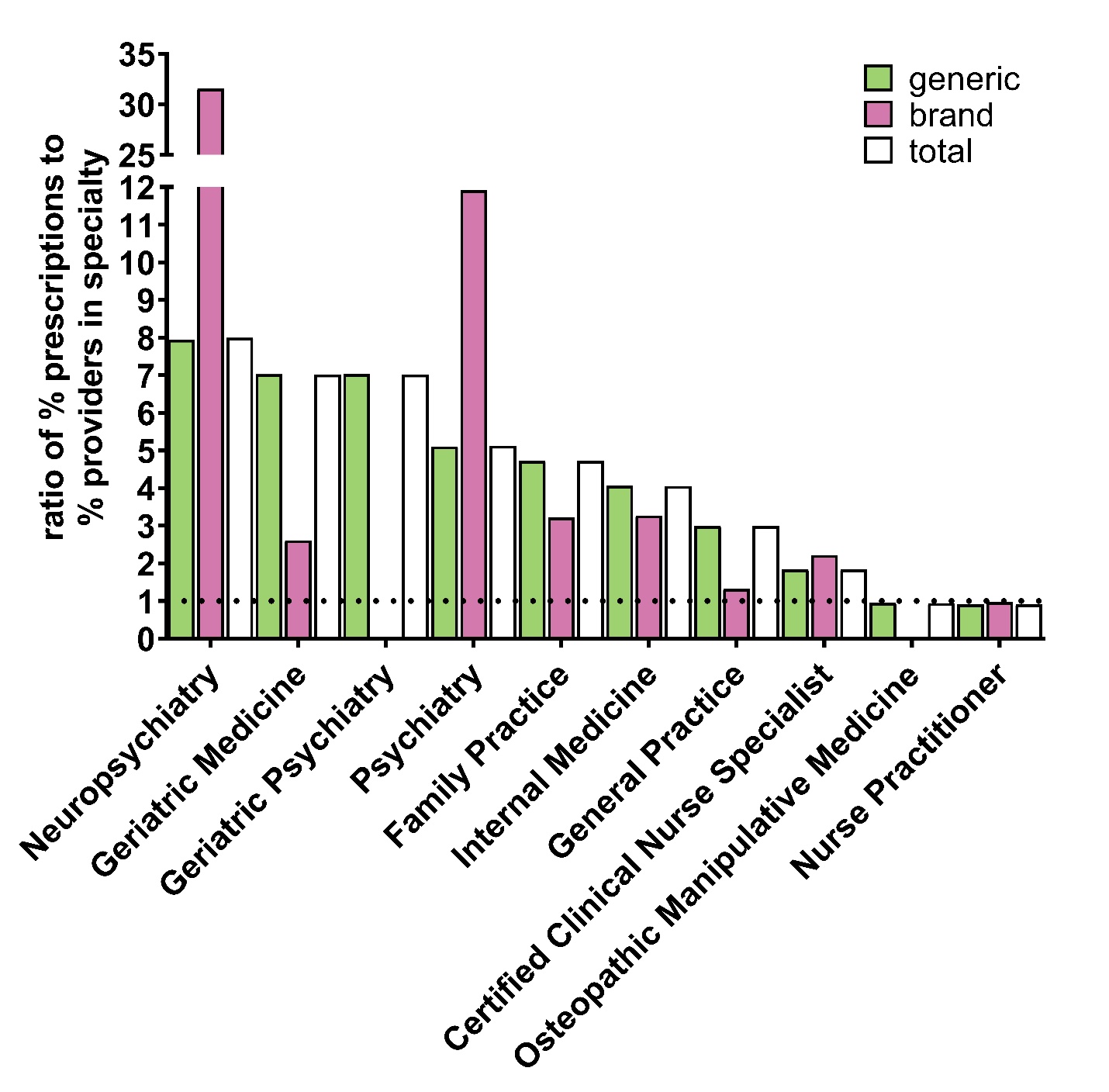
**

**Supplemental Figure 10.** *Specialty types that prescribe the most paroxetine to Medicare Part D enrollees for 2019.* Specialty types that had the highest ratio of percent of paroxetine prescriptions to percent of providers in Medicare who belong to that respective specialty. Dotted line denotes ratio of 1.0.

**Supplemental Table 1**. National Medicare prescription rates and Medicare spending for paroxetine for 2015-2020

|  | prescriptions / thousand enrollees | | | | | | spending ($ / enrollee) | | | | | |
| --- | --- | --- | --- | --- | --- | --- | --- | --- | --- | --- | --- | --- |
|  | generic | Brisdelle | Paxil | Pexeva | total brand | brand + generic | generic | Brisdelle | Paxil | Pexeva | brand | brand + generic |
| 2015 | 121.60 | 0.22 | 0.67 | 0.10 | 1.00 | 122.61 | 105.26 | 1.79 | 8.90 | 1.31 | 12.00 | 117.27 |
| 2016 | 115.91 | 0.18 | 0.62 | 0.08 | 0.88 | 116.80 | 99.15 | 1.64 | 9.58 | 1.15 | 12.37 | 111.52 |
| 2017 | 107.06 | 0.12 | 0.51 | 0.06 | 0.69 | 107.74 | 90.00 | 1.15 | 8.09 | 0.95 | 10.19 | 100.19 |
| 2018 | 96.93 | 0.02 | 0.45 | 0.05 | 0.51 | 97.44 | 85.00 | 0.15 | 7.95 | 0.92 | 9.02 | 94.02 |
| 2019 | 86.58 | 0.01 | 0.31 | 0.04 | 0.36 | 86.94 | 80.98 | 0.11 | 6.20 | 0.80 | 7.11 | 88.09 |
| 2020 | 79.94 | 0.01 | 0.30 | 0.03 | 0.34 | 80.28 | 74.97 | 0.09 | 6.92 | 0.64 | 7.64 | 82.62 |

**Supplemental Table 2.** States’ paroxetine prescriptions per thousand Medicare Part D enrollees for 2015-2020

|  | prescriptions / thousand enrollees | | | | | |
| --- | --- | --- | --- | --- | --- | --- |
|  | 2015 | 2016 | 2017 | 2018 | 2019 | 2020 |
| Alabama | 137.70 | 130.64 | 120.68 | 108.61 | 98.09 | 92.14 |
| Alaska | 178.27 | 142.21 | 117.87 | 108.65 | 74.83 | 70.95 |
| Arizona | 104.93 | 98.87 | 90.02 | 80.88 | 71.68 | 67.28 |
| Arkansas | 137.97 | 134.82 | 127.20 | 116.84 | 105.57 | 97.63 |
| California | 102.94 | 96.44 | 86.82 | 75.95 | 66.00 | 59.40 |
| Colorado | 98.25 | 91.87 | 82.87 | 72.23 | 63.94 | 57.52 |
| Connecticut | 116.29 | 111.73 | 104.01 | 95.08 | 86.20 | 80.80 |
| Delaware | 104.37 | 97.82 | 91.48 | 86.74 | 80.99 | 75.41 |
| District of Columbia | 83.36 | 76.06 | 75.23 | 71.80 | 60.55 | 53.27 |
| Florida | 121.88 | 114.47 | 101.91 | 88.06 | 78.24 | 73.88 |
| Georgia | 134.69 | 127.77 | 120.22 | 109.77 | 96.20 | 86.15 |
| Hawaii | 42.05 | 37.90 | 34.26 | 31.70 | 27.70 | 26.58 |
| Idaho | 119.51 | 108.26 | 96.30 | 89.42 | 80.36 | 72.29 |
| Illinois | 115.59 | 108.55 | 99.24 | 89.08 | 80.03 | 74.78 |
| Indiana | 129.87 | 124.30 | 115.63 | 105.90 | 95.52 | 88.53 |
| Iowa | 152.18 | 144.24 | 130.34 | 118.76 | 105.92 | 97.89 |
| Kansas | 123.89 | 121.34 | 113.78 | 104.46 | 95.54 | 87.69 |
| Kentucky | 194.88 | 195.33 | 182.73 | 165.12 | 143.26 | 132.51 |
| Louisiana | 123.23 | 117.91 | 110.33 | 101.04 | 90.71 | 84.81 |
| Maine | 147.11 | 135.90 | 126.78 | 111.53 | 95.99 | 86.45 |
| Maryland | 109.63 | 105.02 | 99.41 | 93.25 | 85.88 | 77.78 |
| Massachusetts | 152.26 | 142.80 | 133.02 | 121.03 | 104.21 | 95.87 |
| Michigan | 111.80 | 108.94 | 103.47 | 96.32 | 87.61 | 81.55 |
| Minnesota | 121.06 | 118.01 | 108.76 | 98.48 | 86.64 | 79.19 |
| Mississippi | 141.54 | 134.80 | 125.40 | 114.20 | 100.89 | 92.89 |
| Missouri | 143.33 | 137.16 | 128.35 | 118.35 | 105.80 | 99.02 |
| Montana | 116.26 | 110.39 | 102.50 | 91.84 | 81.84 | 74.44 |
| Nebraska | 133.00 | 125.53 | 117.29 | 107.06 | 95.32 | 87.83 |
| Nevada | 95.13 | 87.56 | 80.13 | 72.67 | 64.34 | 59.58 |
| New Hampshire | 138.37 | 129.79 | 121.72 | 109.30 | 97.71 | 89.80 |
| New Jersey | 108.55 | 103.72 | 96.66 | 89.46 | 82.35 | 75.27 |
| New Mexico | 105.24 | 99.79 | 90.95 | 81.46 | 72.96 | 67.47 |
| New York | 121.96 | 117.26 | 111.00 | 103.01 | 93.92 | 87.62 |
| North Carolina | 130.14 | 124.76 | 114.87 | 102.60 | 91.09 | 84.89 |
| North Dakota | 168.70 | 170.67 | 149.95 | 143.32 | 126.34 | 115.04 |
| Ohio | 133.86 | 130.82 | 123.52 | 115.05 | 103.38 | 96.51 |
| Oklahoma | 139.10 | 135.12 | 125.07 | 112.61 | 99.87 | 91.49 |
| Oregon | 120.71 | 110.98 | 99.94 | 88.23 | 77.92 | 70.16 |
| Pennsylvania | 131.44 | 127.04 | 119.69 | 110.70 | 99.91 | 91.96 |
| Rhode Island | 174.94 | 162.10 | 151.34 | 130.02 | 107.96 | 98.37 |
| South Carolina | 117.76 | 114.01 | 106.39 | 97.85 | 88.70 | 80.23 |
| South Dakota | 125.18 | 122.65 | 114.39 | 103.07 | 92.15 | 83.31 |
| Tennessee | 166.52 | 160.26 | 146.02 | 130.55 | 117.22 | 108.81 |
| Texas | 99.19 | 93.93 | 86.01 | 77.75 | 70.16 | 65.95 |
| Utah | 101.03 | 96.56 | 89.25 | 77.13 | 69.12 | 63.04 |
| Vermont | 109.73 | 102.12 | 92.32 | 82.80 | 75.97 | 67.96 |
| Virginia | 126.10 | 118.82 | 109.52 | 100.25 | 89.34 | 81.36 |
| Washington | 111.42 | 103.57 | 93.22 | 83.19 | 74.79 | 68.54 |
| West Virginia | 167.52 | 164.52 | 154.36 | 137.75 | 121.12 | 110.88 |
| Wisconsin | 127.57 | 118.91 | 109.50 | 100.40 | 90.86 | 84.72 |
| Wyoming | 93.21 | 88.79 | 81.38 | 75.87 | 66.56 | 60.69 |

**Supplemental Table 3.** *Specialty types that prescribe the most paroxetine to Medicare Part D enrollees for 2015-2020.* Specialty types that had the highest ratio of percent of paroxetine prescriptions to percent of providers in Medicare who belong to that respective specialty.

|  | 2015 | | | 2016 | | | 2017 | | |
| --- | --- | --- | --- | --- | --- | --- | --- | --- | --- |
|  | generic | brand | total | generic | brand | total | generic | brand | total |
| Certified Clinical Nurse Specialist | 1.25 | 0.00 | 1.25 | 1.35 | 0.00 | 1.34 | 1.42 | 0.00 | 1.41 |
| Dentist | --- | --- | --- | 2.16 | 0.00 | 2.15 | --- | --- | --- |
| Family Practice | 4.54 | 3.04 | 4.54 | 4.63 | 3.02 | 4.63 | 4.64 | 2.70 | 4.63 |
| General Practice | 2.81 | 2.68 | 2.81 | 3.03 | 1.79 | 3.02 | 3.09 | 1.41 | 3.09 |
| Geriatric Medicine | 5.81 | 2.83 | 5.80 | 6.31 | 4.46 | 6.30 | 6.12 | 2.57 | 6.10 |
| Geriatric Psychiatry | 3.90 | 7.85 | 3.92 | 3.94 | 0.00 | 3.92 | 5.68 | 0.00 | 5.67 |
| Internal Medicine | 3.82 | 2.82 | 3.81 | 3.84 | 2.84 | 3.84 | 3.85 | 3.30 | 3.85 |
| Neurology | 0.89 | 1.91 | 0.89 | 0.87 | 1.77 | 0.88 | 0.85 | 1.91 | 0.85 |
| Neuropsychiatry | 6.92 | 74.34 | 7.19 | 6.98 | 33.61 | 7.09 | 7.84 | 45.88 | 7.96 |
| Nurse Practitioner | --- | --- | --- | --- | --- | --- | 0.81 | 0.57 | 0.81 |
| Osteopathic Manipulative Medicine | 0.77 | 0.00 | 0.77 | --- | --- | --- | --- | --- | --- |
| Psychiatry | 4.35 | 8.77 | 4.37 | 4.56 | 10.36 | 4.58 | 4.81 | 11.55 | 4.83 |

|  | 2018 | | | 2019 | | | 2020 | | |
| --- | --- | --- | --- | --- | --- | --- | --- | --- | --- |
|  | generic | brand | total | generic | brand | total | generic | brand | total |
| Certified Clinical Nurse Specialist | 1.63 | 1.17 | 1.63 | 1.84 | 2.22 | 1.84 | 1.93 | 2.50 | 1.93 |
| Dentist | --- | --- | --- | --- | --- | --- | --- | --- | --- |
| Family Practice | 4.65 | 3.05 | 4.64 | 4.72 | 3.22 | 4.72 | 4.73 | 3.17 | 4.72 |
| General Practice | 3.04 | 2.09 | 3.04 | 3.00 | 1.32 | 2.99 | 2.96 | 1.30 | 2.95 |
| Geriatric Medicine | 7.04 | 6.66 | 7.04 | 7.03 | 2.61 | 7.02 | 6.10 | 6.29 | 6.10 |
| Geriatric Psychiatry | 5.41 | 0.00 | 5.40 | 7.03 | 0.00 | 7.02 | 8.21 | 0.00 | 8.19 |
| Internal Medicine | 4.00 | 3.14 | 4.00 | 4.06 | 3.27 | 4.06 | 4.09 | 3.06 | 4.09 |
| Neurology | --- | --- | --- | --- | --- | --- | --- | --- | --- |
| Neuropsychiatry | 8.09 | 19.13 | 8.13 | 7.95 | 31.53 | 7.99 | 9.92 | 57.46 | 10.02 |
| Nurse Practitioner | 0.87 | 0.74 | 0.87 | 0.91 | 0.97 | 0.91 | 1.01 | 1.03 | 1.01 |
| Osteopathic Manipulative Medicine | 0.90 | 0.00 | 0.90 | 0.95 | 0.00 | 0.94 | 0.90 | 0.00 | 0.90 |
| Psychiatry | 4.98 | 12.89 | 5.00 | 5.11 | 11.91 | 5.12 | 5.33 | 12.77 | 5.35 |

--- = not top 10 specialty for that year
